# Facial Reanimation by Masseteric Muscle- Mandibular Periosteum Transfer

**Published:** 2013-01

**Authors:** Ruholah Abbasi

**Affiliations:** Department of Otolaryngology, Besat Hospital, Hamedan, Iran

**Keywords:** Facial Reanimation, Masseteric Muscle, Mandibular periosteum transfer

## Abstract

Permanent facial paralysis is a catastrophic event for involved patients. In long lasting paralysis with severe facial muscles atrophy, masseter muscle transfer is a very good choice. But its greatest problem is postoperative elongation of flap and gradual diminishing of early results and loss of symmetry. This article advocate a new modification for resolving this problem with concomitant elevation of mandibular periosteum with masseter muscle, as a unit for lip and midface elevation.

## INTRODUCTION

Facial paralysis is a profound disfiguring condition with significant psychological and functional consequences. Prior to introduction of surgical reanimation, treatment had been based on medical therapies (botulinum toxin injection), and prosthesis.^1^ Then static surgical reconstruction (slings) was introduced, and after it, use of temporalis and masseteric muscle transfer was popularized. Now nerve grafts and free muscle grafts are choice of facial reanimation for facial paralysis.^2^ This article describes a new modification for masseteric transfer for facial reanimation that can have more constant elevation and contraction results.

## CASE REPORT

**Fig. 1:**
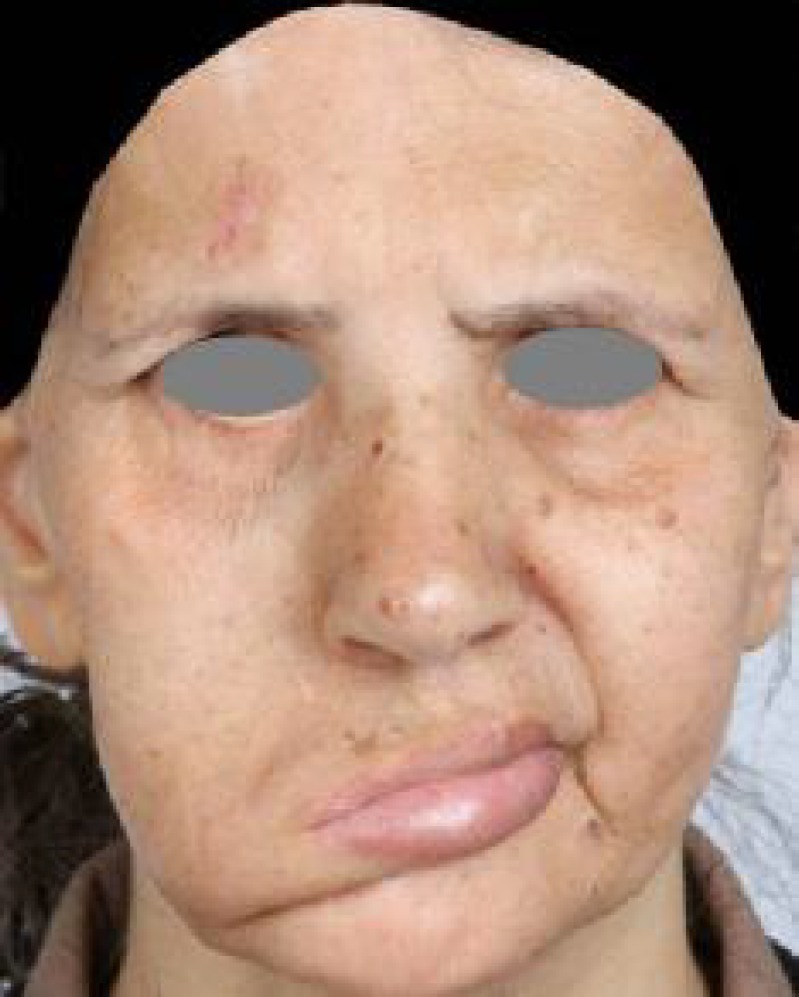
Preoperative severe facial distortion

A 50 year old woman was referred, 15 years after skull based surgery resulting in a right complete facial paralysis. She had atrophy, severe elongation and drooping of right side of face, abundant skin and soft tissue accumulation below the right labial commissure. Severe facial deviation and distortion to normal (left) side were present (Figure 1).

Endoscopic brow elevation was performed, with overcorrection of the paralyzed side (a probable complication is in- creased exposure of eye). Right tarsal strip for the right lower eyelid and tarsorrhaphy were done. A face lift incision was initiated at the root of the helix and continued to the preauricular crease, curved around the earlobe and back and up rotated to the mastoid tip. Considering the history of radiotherapy to the skull base and in- creased risk of skin flap necrosis, a sub-SMAS (superficial musculoaponeurotic system) flap was elevated in preauricular region. Flap elevation was continued to ipsilateral nasolabial fold and philtrum. In doing so a tunnel was created from tragus and angle of mandible to mid portions of upper and lower lips. In conventional masseteric transfer, inferior and middle portions of anterior two thirds of masseter muscle were detached from mandible and were used to elevate oral commissure. Also attachment of masseter to the inferior border of the body of mandible was elevated with masseter to facilitate sutures to the distal part of flap. The most common disadvantage of the conventional procedure was muscle elongation due to the weight of face postoperatively, and some drooping would occur in time. This led to a partial loss of the reconstruction results. In this modification, masseter muscle was elevated in continuity with periosteum of the lateral surface of mandibular ramus as a unit. Small incisions were also made in the centre of vermilion of both lips and connected to the tunnel. The distal end of the masseteric flap was divided to three parts and rotated anteriorly toward central lip incisions. Anterior part was sutured to lower philtrum and vermilion of upper lip, posterior part to lower lip vermilion, and middle part to right oral commissure. One centimeter overcorrection was considered appropriate for reconstruction.

**Fig. 2:**
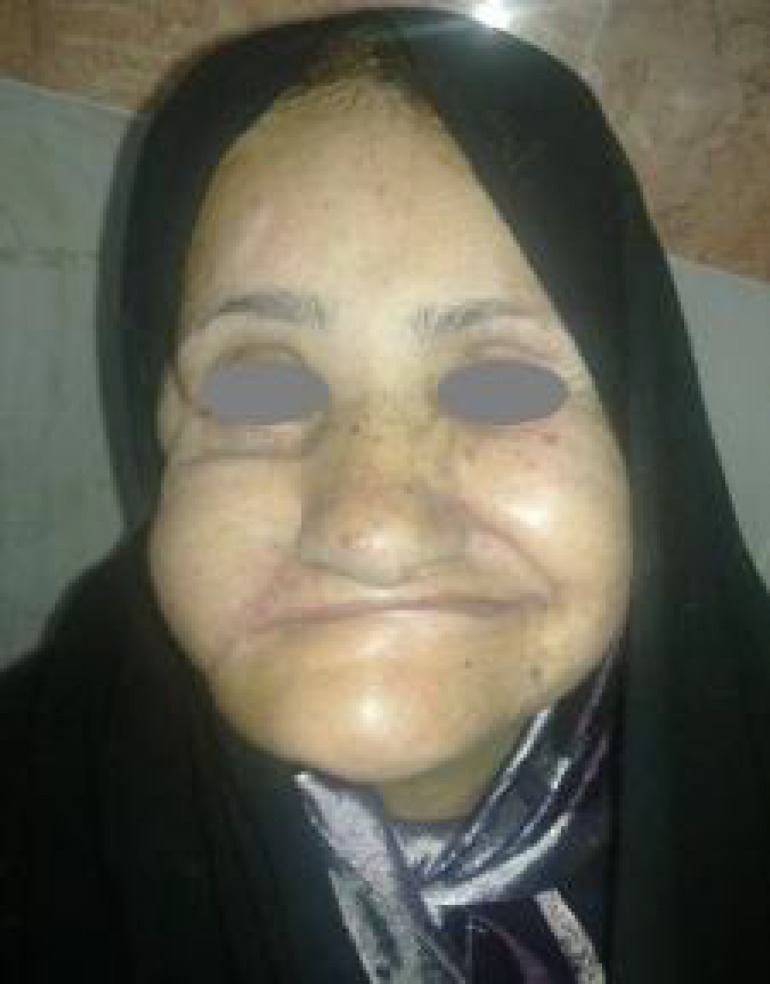
One year after surgery with counter pressure of upper and lower jaws. The patient is edentulous, so some internal distortion of inferior right lower lip behind the upper lip is seen

Massteric transfer with the underlying mandibular periosteum as a unit, augmented the muscle flap and prevented future elongation. It seemed a new modification for masseteric transfer that produced better results with time. Figure 2 shows one year after surgery with counter pressure of upper and lower jaws. The patient was edentulous, so some internal distortion of inferior right lower lip behind the upper lip was seen.

## DISCUSSION

In early facial reanimation (less than two years of initiation of paralysis) for unilateral complete facial paralysis, cross facial nerve graft (CFNG) is modality of choice for innervation that recovers mimetic functions.^1^ But 18 to 24 months after initiation of paralysis, when facial muscle atrophy is irreversible and no fibrillation is found on electromyography (EMG),^2^ free muscle transfer is choice for reanimation and can recover spontaneous smile. Recent articles advocate free muscle transfer neuritized with masseteric nerve (a branch of trigeminal nerve to masseter muscle), because of the accessibility of this nerve, rapid symmetry and forty percent spontaneous smile acquiring.^1^^,^^3^^,^^4^^,^^5^^,^^6^

We believe although neuritization of a free muscle with masseter nerve can provide a very good result, use of massetric muscle itself and its nerve, without transection between muscle and nerve, can be a better choice and achieves better results.

## CONFLICT OF INTEREST

The authors declare no conflict of interest.

## Footenote

Please cite this paper as:

Abbasi R. Facial Reanimation by Masseteric Muscle-Mandibular Periosteum Transfer. World J Plast Surg 2013;2(1): 44-46.
